# Supplementary material for: Tackling realistic Li+ flux for high-energy lithium metal batteries
Source: Nat Commun. 2022 Sep 16;13:5431. doi: 10.1038/s41467-022-33151-w (PMC9481556; doi:10.1038/s41467-022-33151-w)
Supplement: Supplementary file 1 — Supplementary Information File [file 41467_2022_33151_MOESM1_ESM.pdf]

Supplementary Information

Tackling realistic Li^+ flux for high-energy lithium metal batteries

Shuoqing Zhang[1+], Ruhong Li[1+], Nan Hu[2+], Tao Deng[3], Suting Weng[4], Zunchun Wu[1], Di Lu[1], Haikuo Zhang[1], Junbo Zhang[1], Xuefeng Wang[4], Lixin Chen[1,5], Liwu Fan[2,6*], and Xiulin Fan[1*]

1 State Key Laboratory of Silicon Materials, School of Materials Science and Engineering, Zhejiang University, Hangzhou 310027, China.

2 State Key Laboratory of Clean Energy Utilization, School of Energy Engineering, Zhejiang University, Hangzhou, 310027, China.

3 Department of Chemical and Biomolecular Engineering, University of Maryland, College Park, MD 20742, USA.

4 Beijing National Laboratory for Condensed Matter Physics, Institute of Physics, Chinese Academy of Sciences, Beijing 100190, China.

5 Key Laboratory of Advanced Materials and Applications for Batteries of Zhejiang Province, Hangzhou 310013, China.

6 Key Laboratory of Clean Energy and Carbon Neutrality of Zhejiang Province, Hangzhou 310027, China.

[+]These authors contributed equally to this work.

*Corresponding authors: liwufan@zju.edu.cn, xlfan@zju.edu.cn

Table of Contents

Table S1. Formula of BE and fluorine-rich electrolytes.

Table S2. Detailed information of the dual-halide electrolytes.

Table S3. Necessary parameters in the COMSOL simulation.

Figure S1. Li^+ concentration distribution in electrolyte.

Figure S2. Li^+ concentration distribution considering high and low mobility region.

Figure S3. Representative voltage-time plots of Li||Li cells.

Figure S4. Basic properties of the investigated electrolytes.

Figure S5. HOMO and LUMO energy for currently used Li salts and reagents.

Figure S6. Optical images of Li deposits and cathode shells.

Figure S7. Simulation of Li^+ migration in 6 M HCE electrolyte.

Figure S8. Simulation of Li^+ migration in 1.3 M LDC electrolyte.

Figure S9. Li^+ clusters in 1.3 M LDC electrolyte.

Figure S10. Evolution of interphase between LMA and 1.3 M LDC electrolyte.

Figure S11. XPS spectra of SEI formed in 1 M BE.

Figure S12. The configuration and binding energy landscape of Li_2O.

Figure S13. The configuration and binding energy landscape of Li_2CO_3.

Figure S14. The configuration and binding energy landscape of α-Li_3N.

Figure S15. The configuration of LiF.

Figure S16. Atomic ratio of F and Cl in SEI with different sputtering time.

Figure S17. Comparison of different $LiF_{1-x}Cl_x$ configurations.

Figure S18. CE of Li||Cu cells with different electrolytes.

Figure S19. Overpotentials of Li||Cu cells in 1.3 M LDC with different cycles.

Figure S20. SEM images of the Li deposits.

Figure S21. Comparison of optical images of a Li foil soaking in DCE for 1 week

Figure S22. EIS tests of Li||Li cells.

Figure S23. The corrosivity of different electrolytes to Al foils.

Figure S24. Voltage profiles of LMBs.

Figure S25. Cycle performance of Li||NCM811 cells tested in 2.2 M LiFSI/DMC-DCE electrolyte.

Figure S26. Cycle performance of Li||NCM811 cells.

Figure S27. Cycle performance of Li||LCO batteries with 1.3 M LDC electrolyte.

Figure S28. GITT curves of NCM811 in 6 M HCE and 1.3 M LDC.

Figure S29. Rate capabilities of different electrolytes.

Figure S30. Comparison of anode-free pouch cells with 1.3 M LDC before cycle and after cycle.

Figure S31. SEM images of NCM811 particles.

Figure S32. Surface chemistry of NCM811 cycled in 1 M BE electrolyte.

Figure S33. Evolution of impedance in Li||NCM811 cells.

Table S1 Formula of BE and fluorine-rich electrolytes

Salt	Solvent	Abbreviation	Ref.
1 M $LiPF_6$	ethylene carbonate/dimethyl carbonate (EC/DMC)	BE	
6 M LiFSI	Dimethoxyethane (DME)	HCE	1
LiFSI	DME/1,1,2,2-tetrafluoroethyl-2,2,3,3-tetrafluoropropyl ether	DME-TTE	2
LiFSI	DMC-TTE	DMC-TTE	3
LiFSI	DME/Fluorobenzene	DME-FB	4

Table S2 Detailed information of the dual-halide electrolytes

Electrolytes	Molar ratio	Volume ratio	Density ($g\ cm^{-3}$)	Molarity ($mol\ L^{-1}$)
LiFSI/DME-DCE	0.949/1/6	1/4.531	1.193	1.3
LiFSI/DME-PhCl	0.806/1/2	1/1.951	1.270	2.5
LiFSI/DME-TCE	0.811/1/2	1/2.026	1.495	2.4
LiFSI/DMC-DCE	0.696/1/2	1/1.904	1.312	2.2

Abbreviation:

Lithium bis(fluorosulfonyl)imide LiFSI

Lithium hexafluorophosphate $LiPF_6$

Ethylene carbonate EC

Dimethyl carbonate DMC

Dimethoxyethane DME

1,1,2,2-tetrafluoroethyl-2,2,3,3-tetrafluoropropyl ether TTE

Fluorobenzene FB

1,1,2,2-Tetrachloroethane TCE

1,2-dichloroethane DCE

Chlorobenzene PhCl

Table S3 Necessary parameters in the COMSOL simulation

Name	HCE	LDC
Electrolyte diffusion coefficient ($cm^2\ s^{-1}$)	3.5×10^{-6}	1.5×10^{-5}
Electric conductivity of electrolyte ($mS\ cm^{-1}$)	0.68	4.30
Transfer number	0.38	0.60
SEI thickness (nm)	10	8.5
Electric conductivity of high mobility SEI ($mS\ cm^{-1}$)	6×10^{-8}	6×10^{-8}
Electric conductivity of low mobility SEI ($mS\ cm^{-1}$)	6×10^{-9}	5.7×10^{-8}
Electrolyte thickness (μm)	25	
SEI width of high or low mobility (μm)	30	
Initial concentration (M)	1	
Temperature (K)	293	
Relative equilibrium potential (V)	0	
Anode potential (V)	0.2	0.1
Alpha	0.5	
Beta	0.5	

Supplementary Note 1 The rationales of equivalent circuit for Li^+ diffusion across electrolyte and SEI

(i) Linear distribution of electrolyte concentration

Considering a representative Li||Li cell with the finite internal electrode distance L (~25 μm) and assuming a same SEI thickness δ (~10 nm) on both anode and cathode, the Li^+ concentration in the electrolyte c_E ($0<x<L$) and SEI region c_S ($L<x<L+\delta$) is governed by diffusion equations, as given by

$$\begin{cases} \dfrac{\partial c_S}{\partial t} = D_S \dfrac{\partial^2 c_S}{\partial x^2}, \quad 0 < x < \delta, \ L+\delta < x < L+2\delta \\ \dfrac{\partial c_E}{\partial t} = D_E \dfrac{\partial^2 c_E}{\partial x^2}, \quad \delta < x < L+\delta \end{cases}$$
$$c_E = c_S, \quad x = \delta \,\&\, x = L+\delta \tag{1}$$
$$c_E = c_S = c_0, \quad t = 0$$

where D_E (~10^{-6} cm^2 s^{-1}) and D_S (~10^{-9} cm^2 s^{-1} for high mobility or 10^{-10} cm^2 s^{-1} for low mobility region) are the apparent Li^+ diffusion coefficients in the electrolyte and SEI, respectively, c_0 is the initial Li^+ concentration. Hence, the limit current density j_{lim} can be easily obtained by transforming Eq. (1) into steady equation and setting $c_S = 0$ ($x = L+\delta$) and $c_S = 2c_0$ ($x = 0$), i.e.

$$j_{lim} = \frac{2nFc_0}{\dfrac{L}{D_E} + \dfrac{2\delta}{D_S}}, \tag{2}$$

where n is the stoichiometric number of electrons consumed in the electrode reaction (e.g. 1 for reduction of Li^+), F is the Faraday's constant (96485 C mol^{-1}). $\dfrac{L}{D_E} + \dfrac{2\delta}{D_S}$ is the comprehensive resistance of LMB. If applying a constant current density j ($< j_{lim}$), the Li^+ concentration distribution can meet a steady linear profile in both electrolyte

and SEI region after initialization stage. As shown in Figure S1, the dimensionless initialization time t^*_{ini} in electrolyte (Figure S1a and S1b) and SEI region (Figure S1c and S1d) can be numerically obtained, respectively, by solving the following dimensionless diffusion equations

$$\begin{aligned} \frac{\partial c_{\text{E}}^*}{\partial t^*} &= \frac{D_{\text{E}} t_{\text{c}}}{L^2} \frac{\partial^2 c_{\text{E}}^*}{\partial (x_{\text{E}}^*)^2}, \quad 0 < x_{\text{E}}^* < 1 \\ \frac{\partial c_{\text{S}}^*}{\partial t^*} &= \frac{D_{\text{S}} t_{\text{c}}}{\delta^2} \frac{\partial^2 c_{\text{S}}^*}{\partial (x_{\text{S}}^*)^2}, \quad 0 < x_{\text{S}}^* < 1. \end{aligned} \tag{3}$$

with the characteristic concentration c_0, characteristic length L for electrolyte, characteristic length δ for SEI and proper self-valued characteristic time t_{c}.

It was shown that the dimensionless initialization time t^*_{ini} of both electrolyte and SEI region is only affected by diffusion coefficients D_{E} (or D_{S}) and characteristic length L. In this work, $t^*_{\text{ini}} \sim 0.2$ in electrolyte and $t^*_{\text{ini}} \sim 0.4$ in SEI, which is equivalent to t_{ini} $\sim$ 4 s in electrolyte and to $t_{\text{ini}} \sim 8\times10^{-3}$ or 8×10^{-4} s in SEI. Both initialization times are far less than complete discharging time t_{dis} at the level of several hours. This result demonstrates that once the Li deposition is initiated, the Li^+ concentration inside the battery will quickly reaches a linear distribution and remains steady over the following deposition process.

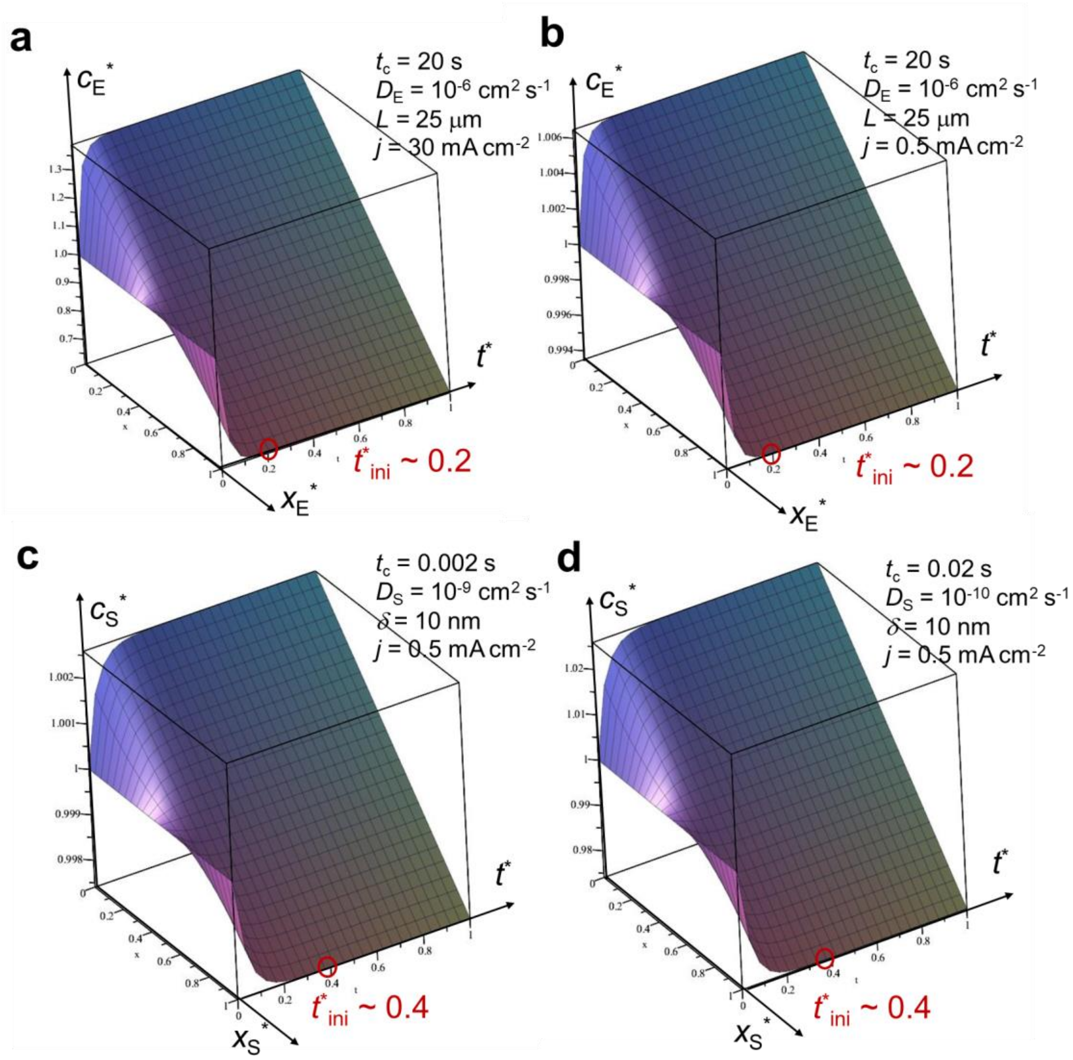

Figure S1. Li^+ concentration distribution in electrolyte. The evolution of Li^+ concentration distribution in electrolyte with L = 25 μm and $D_E = 10^{-6}$ cm² s⁻¹ at current densities of (a) j = 30 mA cm⁻² and (b) j = 0.5 mA cm⁻², as well as in SEI with δ =10 nm at current density j = 0.5 mA cm⁻² when (c) $D_S = 10^{-9}$ cm² s⁻¹ and (d) $D_S = 10^{-10}$ cm² s⁻¹.

(ii) Parallel Li^+ flux along high and low mobility pathways

Given the above analysis, linear distribution of electrolyte concentration is satisfied during the whole Li deposition process. In terms of co-existing high and low mobility regions in SEI, two parallel linear concentration distributions are established like Figure S2a where mass transfer resistance in electrolyte and SEI are connected tandemly, i.e.

$X_{E,l} + 2X_{S,l} \sim \frac{L}{D_E} + \frac{2\delta}{D_{S,l}}$ for low mobility pathway and $X_{E,h} + 2X_{S,h} \sim \frac{L}{D_E} + \frac{2\delta}{D_{S,h}}$ for high mobility pathway when assuming a uniform SEI thickness. By substituting $L \sim 25$ μm, $\delta \sim 10$ nm, $D_E \sim 10^{-6}$ cm^2 s^{-1}, $D_{S,h} \sim 10^{-9}$ cm^2 s^{-1} and $D_{S,l} \sim 10^{-10}$ cm^2 s^{-1} as representative values into following two equations describing current density conservation

$$\begin{cases} nFD_E \frac{\Delta c_{E,l}}{L} = D_{S,l} \frac{\Delta c_{S,l}}{\delta} nF \\ nFD_E \frac{\Delta c_{E,h}}{L} = D_{S,h} \frac{\Delta c_{S,h}}{\delta} nF \end{cases}, \quad (4)$$

the concentration profiles are obtained in Figure S2b, which shows the concentration distribution in electrolyte is influenced by corresponding SEI resistance and results in a higher Li^+ flux $\boldsymbol{J}_h$ along high mobility pathway than that flux $\boldsymbol{J}_l$ along low mobility pathway. The difference of concentration flux $\boldsymbol{J}_h$- $\boldsymbol{J}_l$ is responsible for the uneven Li deposition and dendritic Li growth.

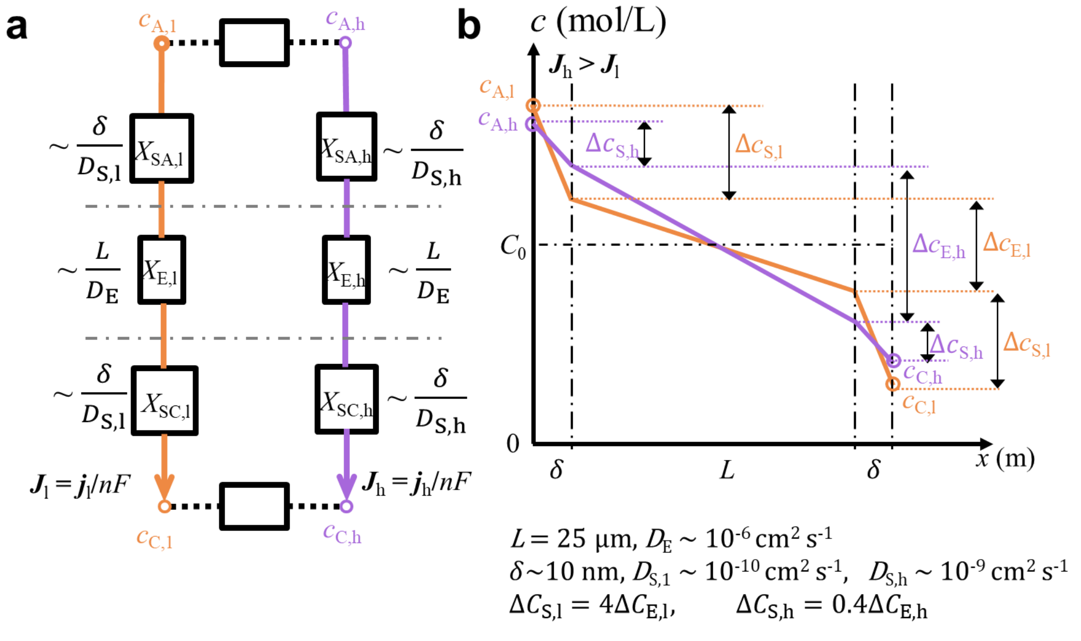

Figure S2. Li^+ concentration distribution considering high and low mobility region. (a) Schematic

diagram of mass transfer resistance and (b) concentration profile along high and low mobility ways assuming diffusion coefficient $D_{S,l}$ ~ 10^{-10} cm^2 s^{-1} in low mobility region and $D_{S,h}$ ~ 10^{-9} cm^2 s^{-1} in high mobility region.

Supplementary Note 2 The expression of $Q_{\text{Li-residue}}$

Equivalently assuming a same thickness δ (~10 nm) and diffusion coefficient of SEI on both anode and cathode, the electric resistances of corresponding low and high Li^+ mobility regions are given based on the series connection between SEI and electrolyte, respectively, by

$$R_{\mathrm{E,l}}+2R_{\mathrm{S,l}}\sim\frac{L}{\theta AD_{\mathrm{E}}}+\frac{2\delta}{\theta AD_{\mathrm{S,l}}}\tag{5}$$

$$R_{\mathrm{E,h}}+2R_{\mathrm{S,h}}\sim\frac{L}{(1-\theta)AD_{\mathrm{E}}}+\frac{2\delta}{(1-\theta)AD_{\mathrm{S,h}}}\tag{6}$$

The deposition currents I and total current density j can be described by:

$$I_{\mathrm{l}}+I_{\mathrm{h}}=j_{\mathrm{l}}\theta A+j_{\mathrm{h}}(1-\theta)A=jA=I\tag{7}$$

where A is the area of Li foil, and θ means the proportion of low mobility region.

According to Ohm law, the high current I_{h} and low current I_{l} can be deduced as

$$I_{\mathrm{l}}=\frac{\dfrac{L}{(1-\theta)D_{\mathrm{E}}}+\dfrac{2\delta}{(1-\theta)D_{\mathrm{S,h}}}}{\dfrac{L}{(1-\theta)D_{\mathrm{E}}}+\dfrac{2\delta}{(1-\theta)D_{\mathrm{S,h}}}+\dfrac{L}{\theta D_{\mathrm{E}}}+\dfrac{2\delta}{\theta D_{\mathrm{S,l}}}}I\tag{8}$$

$$I_{\mathrm{h}}=\frac{\dfrac{L}{\theta D_{\mathrm{E}}}+\dfrac{2\delta}{\theta D_{\mathrm{S,l}}}}{\dfrac{L}{(1-\theta)D_{\mathrm{E}}}+\dfrac{2\delta}{(1-\theta)D_{\mathrm{S,h}}}+\dfrac{L}{\theta D_{\mathrm{E}}}+\dfrac{2\delta}{\theta D_{\mathrm{S,l}}}}I\tag{9}$$

Hence, the residual Li $Q_{\text{Li-residue}}$ due to difference between high current density j_{h} and low current density j_{l} can be derived

$$Q_{\text{Li-residue}}=\frac{t_{\mathrm{dis}}\theta A(j_{\mathrm{h}}-j_{\mathrm{l}})}{nF}=\frac{At_{\mathrm{dis}}j}{nF}\frac{1-\dfrac{D_{\mathrm{S,l}}}{D_{\mathrm{S,h}}}}{\dfrac{(1-\theta)}{\theta}+\dfrac{D_{\mathrm{S,l}}}{D_{\mathrm{S,h}}}+\dfrac{LD_{\mathrm{S,l}}}{2\theta\delta D_{\mathrm{E}}}}\tag{10}$$

where t_{dis} is the total deposition time, n is the stoichiometric number of electrons consumed in the electrode reaction (e.g. 1 for reduction of Li^{+}), and F is the Faraday's constant (96485 C mol^{-1}).

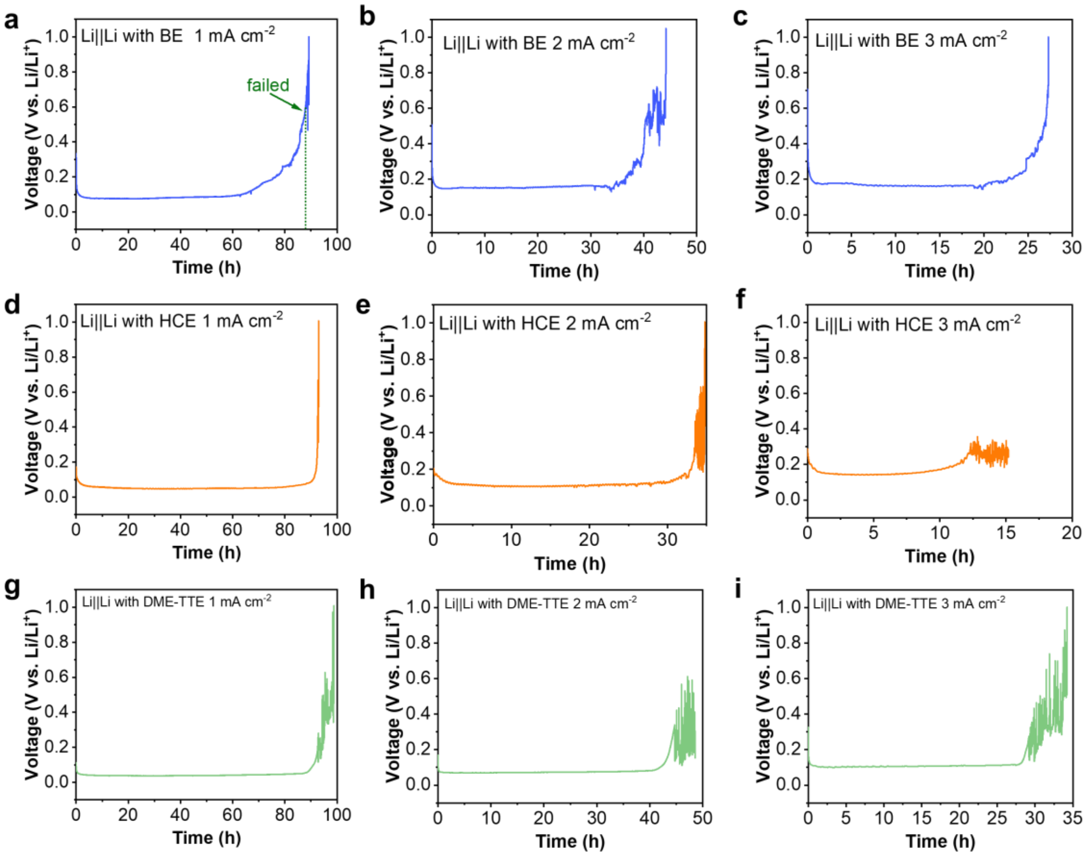

Figure S3. Representative voltage-time plots of Li||Li cells. Li||Li cells tested in (a, b, c) 1 M $LiPF_6$/EC-DMC (BE), (d, e, f) 6 M LiFSI/DME (HCE) and (g, h, i) LiFSI/DME-TTE at 1, 2 and 3 mA cm^{-2}, respectively. 450 μm Li foils were used, corresponding to 92.7865 mAh cm^{-2}.

Li||Li cells with various electrolytes were charged at different current densities until the cell failed. The corresponding time is noted as the t_{dis}. Here, BE, HCE and DME-TTE were taken as examples. The Q_{total} = 92.7865 mA h cm^{-2}. $Q_{deposit}$ is the product of current density (j) and t_{dis}.

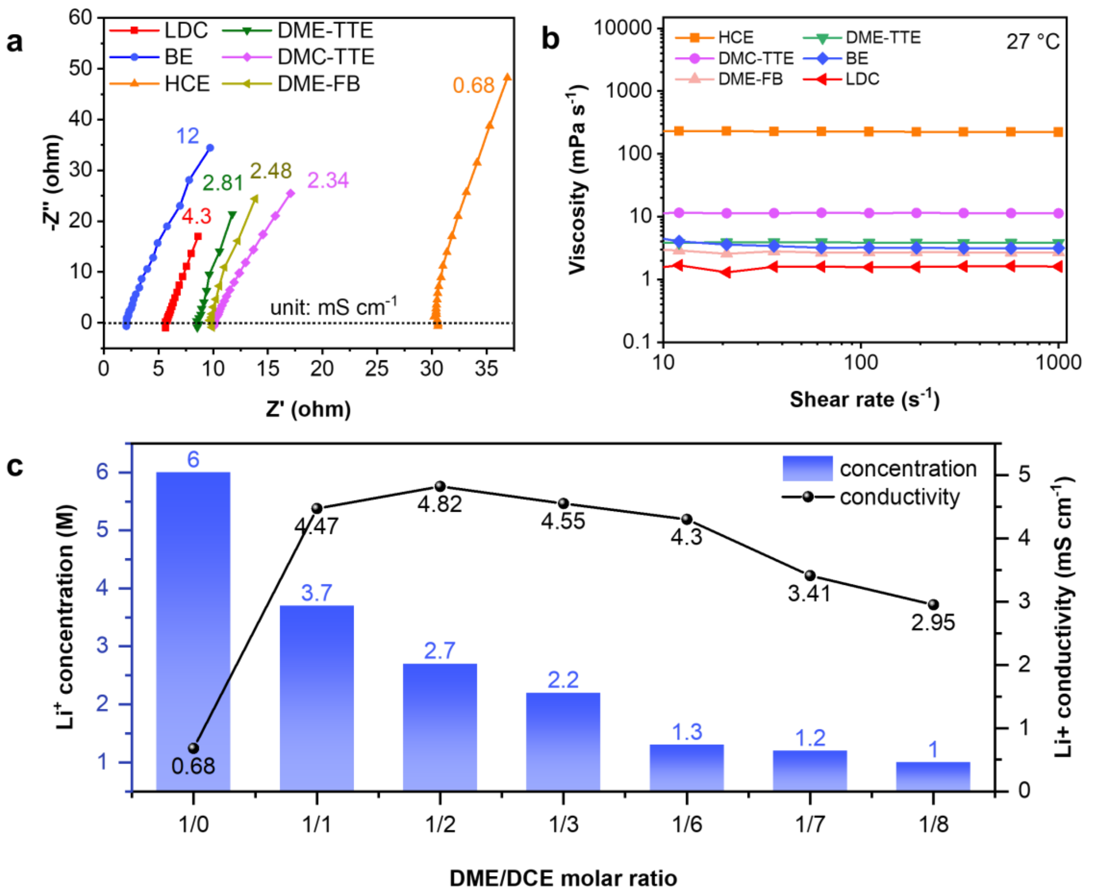

Figure S4. Basic properties of the investigated electrolytes. (a) Li^+ conductivity and (b) viscosity of 1 M $LiPF_6$/EC-DMC (BE), 1.3 M LiFSI/DME-DCE (1.3 M LDC), 6 M LiFSI/DME (HCE) electrolytes, LiFSI/DME-TTE[2], LiFSI/DMC-TTE[3] and LiFSI/DME-FB[4]. (c) Variation of Li^+ concentration and conductivity with the DME/DCE molar ratio.

Figure S4c displays the variation of Li^+ concentration and conductivity with the DME/DCE molar ratio. 1.3 M LDC electrolyte was selected for further investigation due to its high conductivity and comparable concentration to commercial electrolyte.

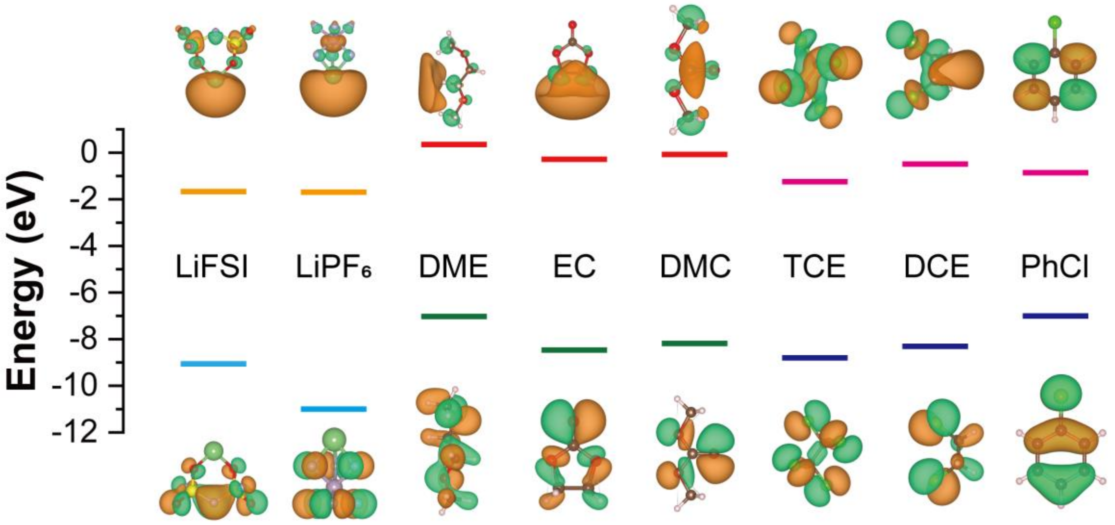

Figure S5. HOMO and LUMO energy for currently used Li salts and reagents.

Abbreviation:

Lithium bis(fluorosulfonyl)imide LiFSI

Lithium hexafluorophosphate $LiPF_6$

Dimethoxyethane DME

1,1,2,2-Tetrachloroethane TCE

1,2-dichloroethane DCE

Chlorobenzene PhCl

According to the molecular orbital energy levels, FSI^- anions are expected to preferentially decompose and generate LiF species, accompanying the reduction of Cl-containing reagents. Therefore, the $LiF_{1-x}Cl_x$-rich SEI can in situ cover the LMA without compromising the mechanical stability to Li dendrites.

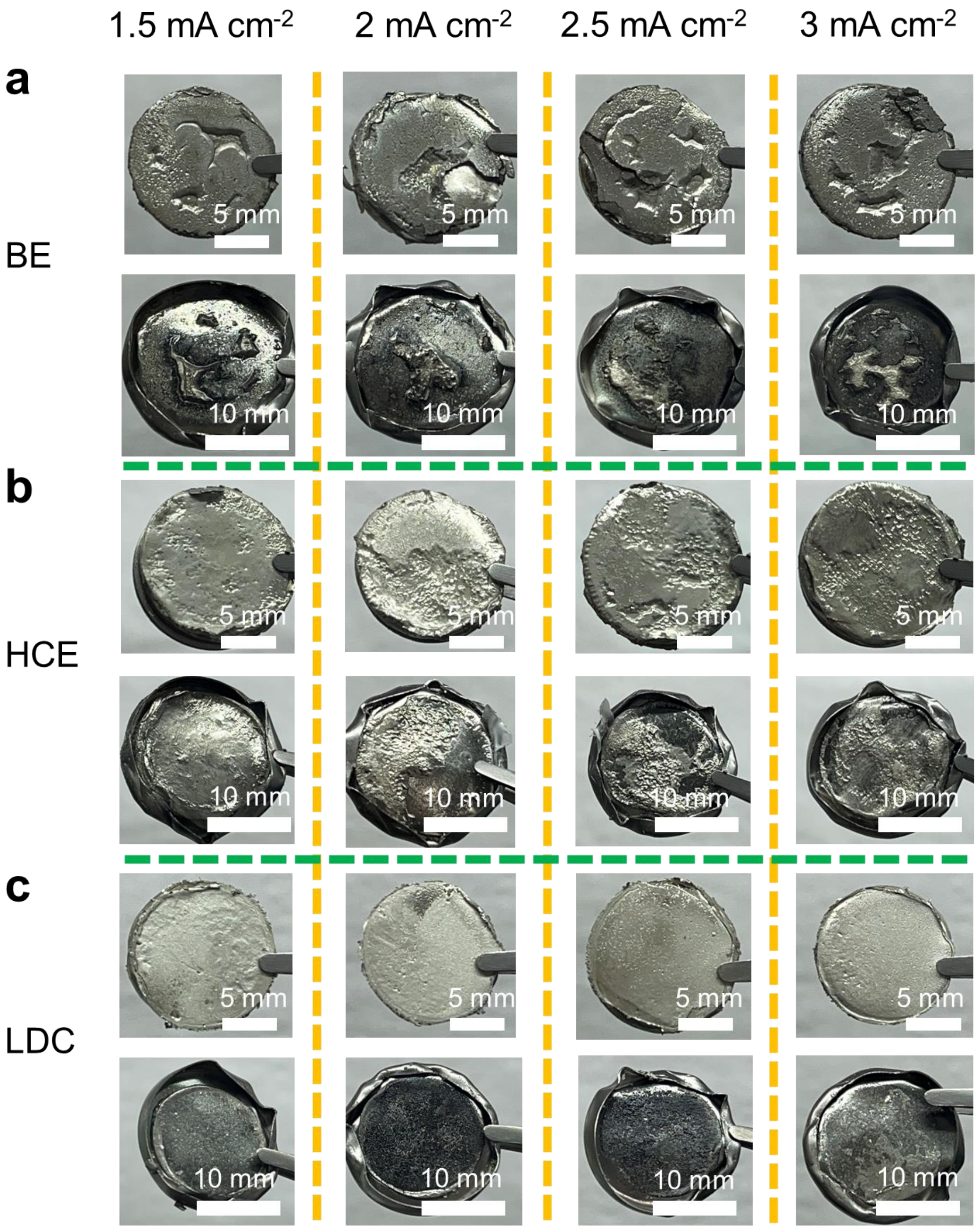

Figure S6. Optical images of Li deposits and cathode shells. Li||Li cells with (a) 1 M $LiPF_6$/EC-DMC (BE), (b) 6 M LiFSI/DME (HCE) and (c) 1.3 M LiFSI/DME-DCE (1.3 M LDC) electrolytes.

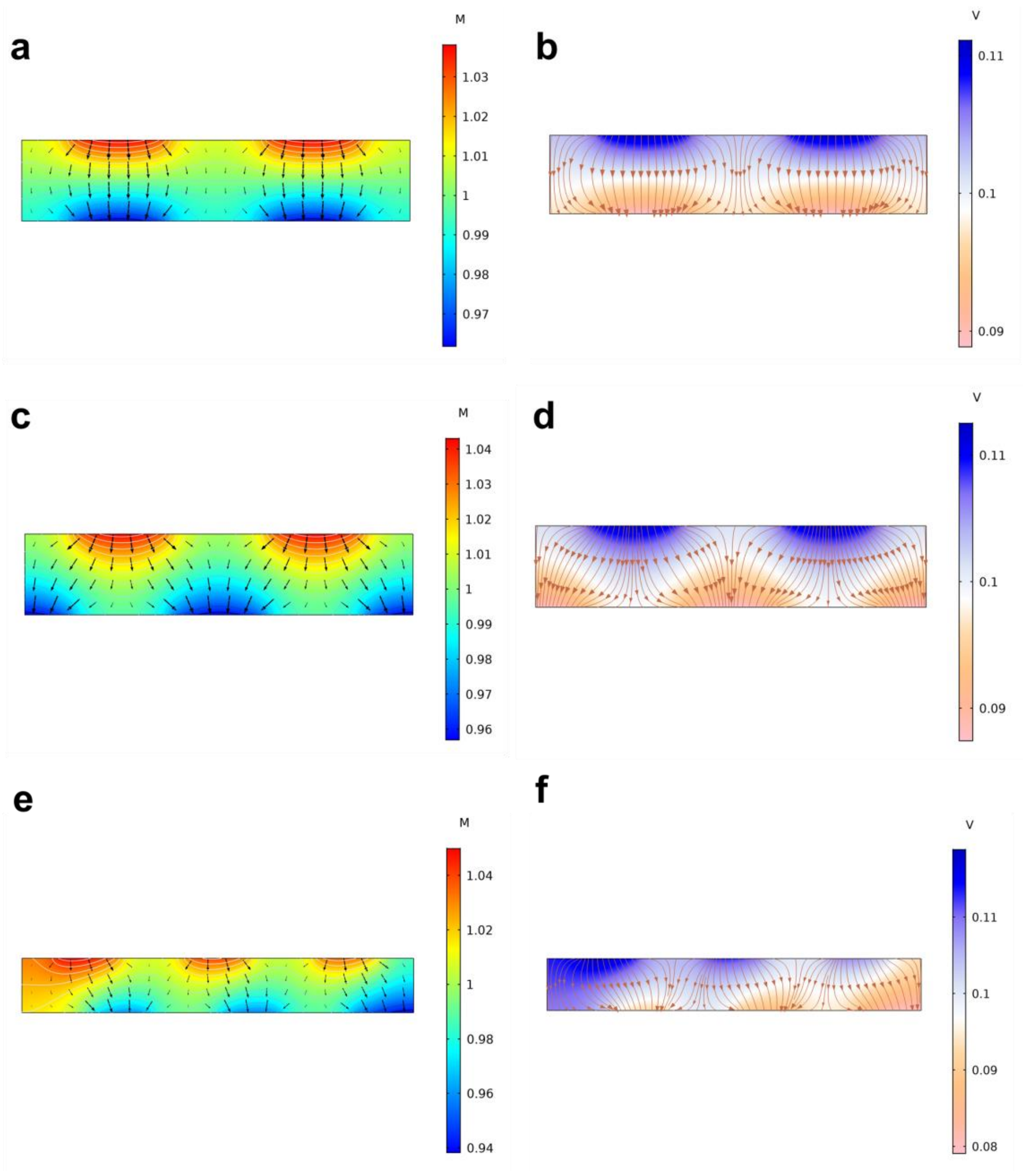

Figure S7. Simulation of Li^+ migration in 6 M LiFSI/DME (HCE) electrolyte. Li^+ concentration (a, c, e) and potential distribution (b, d, f) across 6 M HCE electrolyte with different SEI arrangement.

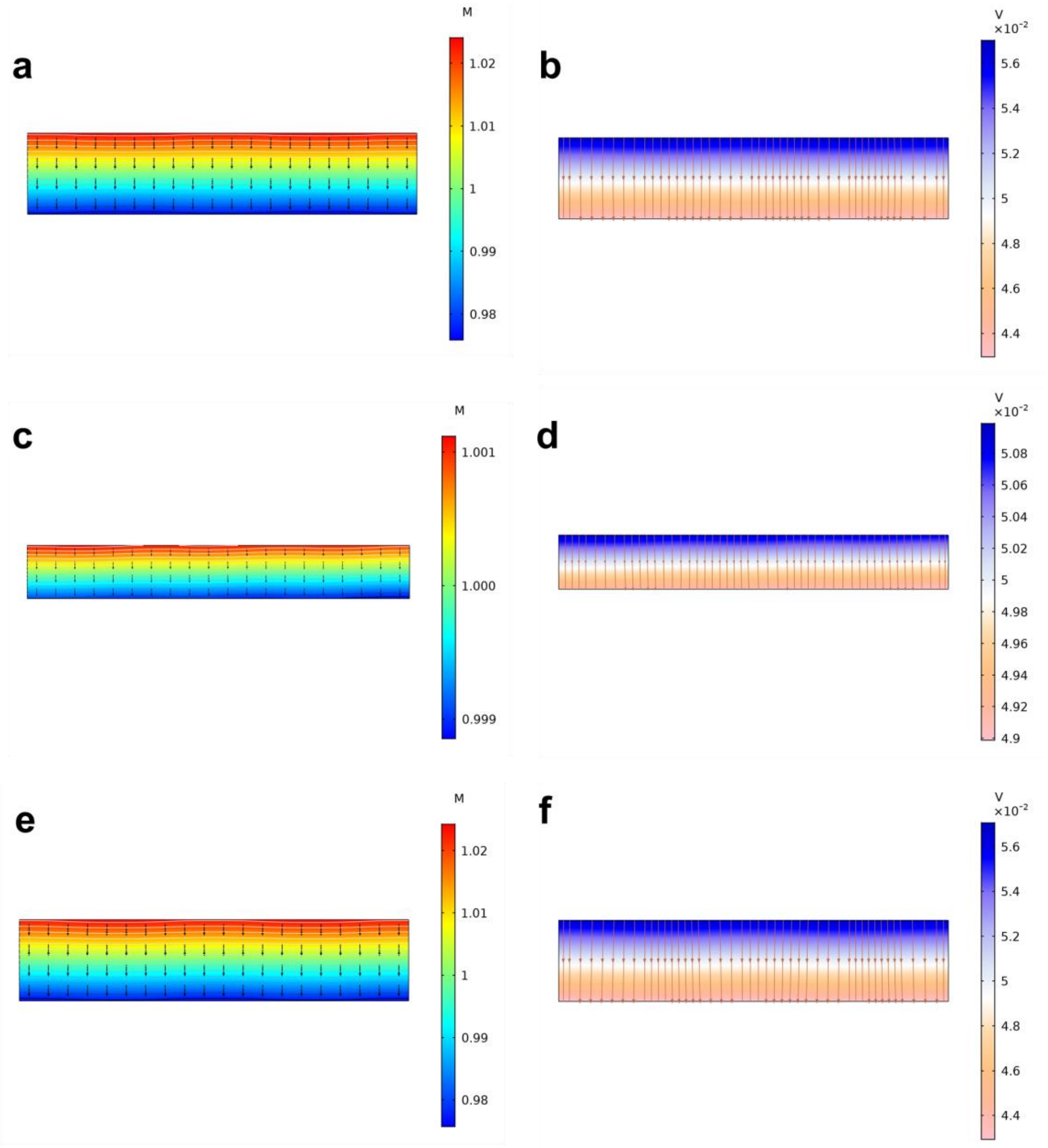

Figure S8. Simulation of Li^+ migration in1.3 M LiFSI/DME-DCE (1.3 M LDC) electrolyte. Li^+ concentration (a, c, e) and potential distribution (b, d, f) across 1.3 M LDC electrolyte with different SEI arrangement.

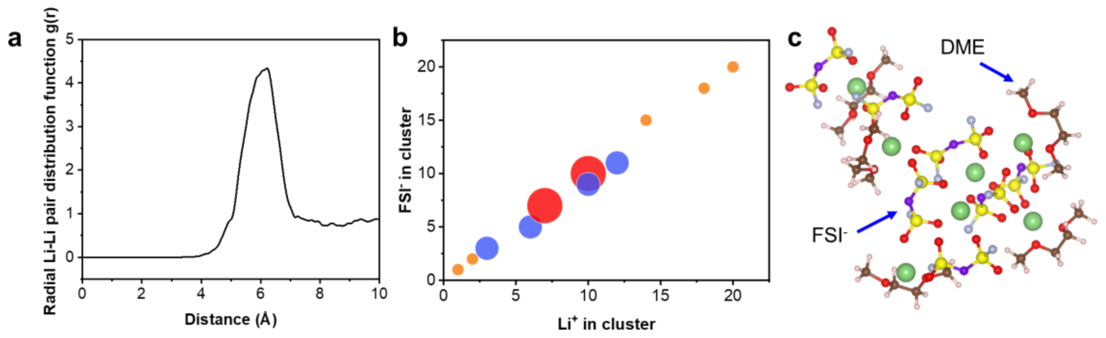

Figure S9. Li^+ clusters in 1.3 M LDC electrolyte. (a) Radial Li-Li pair distribution function, (b) statistical results of the ion clusters of 1.3 M LDC electrolyte. (c) Representative ion clusters consisting of 6 Li^+ ions and 6 FSI^- anions.

Figure S9a displays the radial Li-Li pair distribution function. The sharp peak at 6 Å indicates the large ion clusters of AGGs. Figure S9b exhibits the statistical results of ion clusters. The ion clusters consisting of 6 Li^+/6 FSI^- and 10 Li^+/10 FSI^- account for the largest proportion, confirming the domination of AGGs in 1.3 M LDC electrolyte. Figure S9c illustrates a representative ion cluster including 6 Li^+ ions and 6 FSI^- anions.

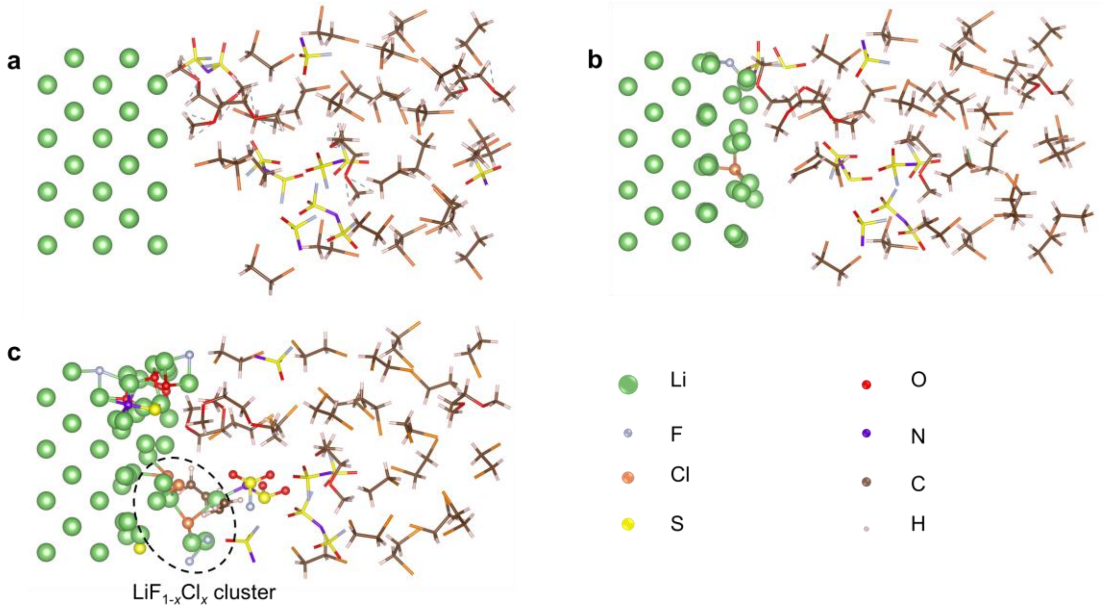

Figure S10. Evolution of interphase between LMA and 1.3 M LDC electrolyte: (a) 0 fs, (b) 200 fs and (c) 1000 fs.

Figure S10 illustrates the ab initio MD results of interphase between LMA and 1.3 M LDC electrolyte, where line model represents the unreacted components and ball-stick model represents the reduction products. After 1000 fs simulation, the $LiF_{1-x}Cl_x$ cluster can be observed at the LMA surface.

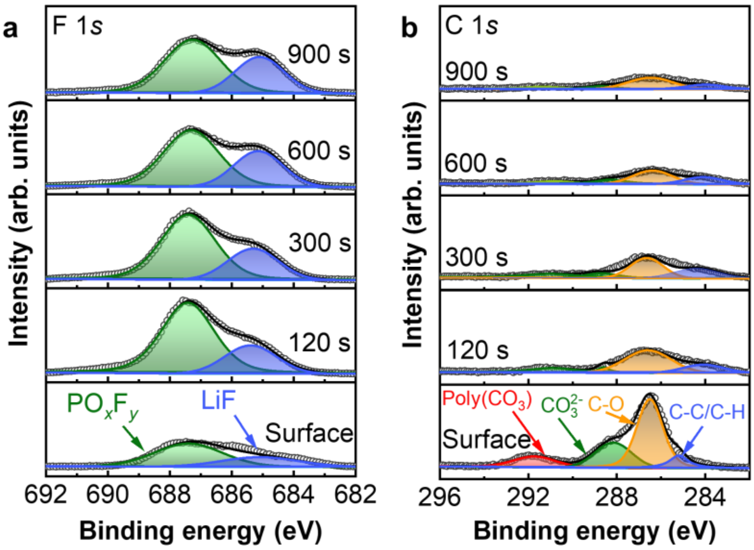

Figure S11. XPS spectra of SEI formed in 1 M $LiPF_6$/EC-DMC (BE): (a) F 1*s* spectra and (b) C 1*s* spectra.

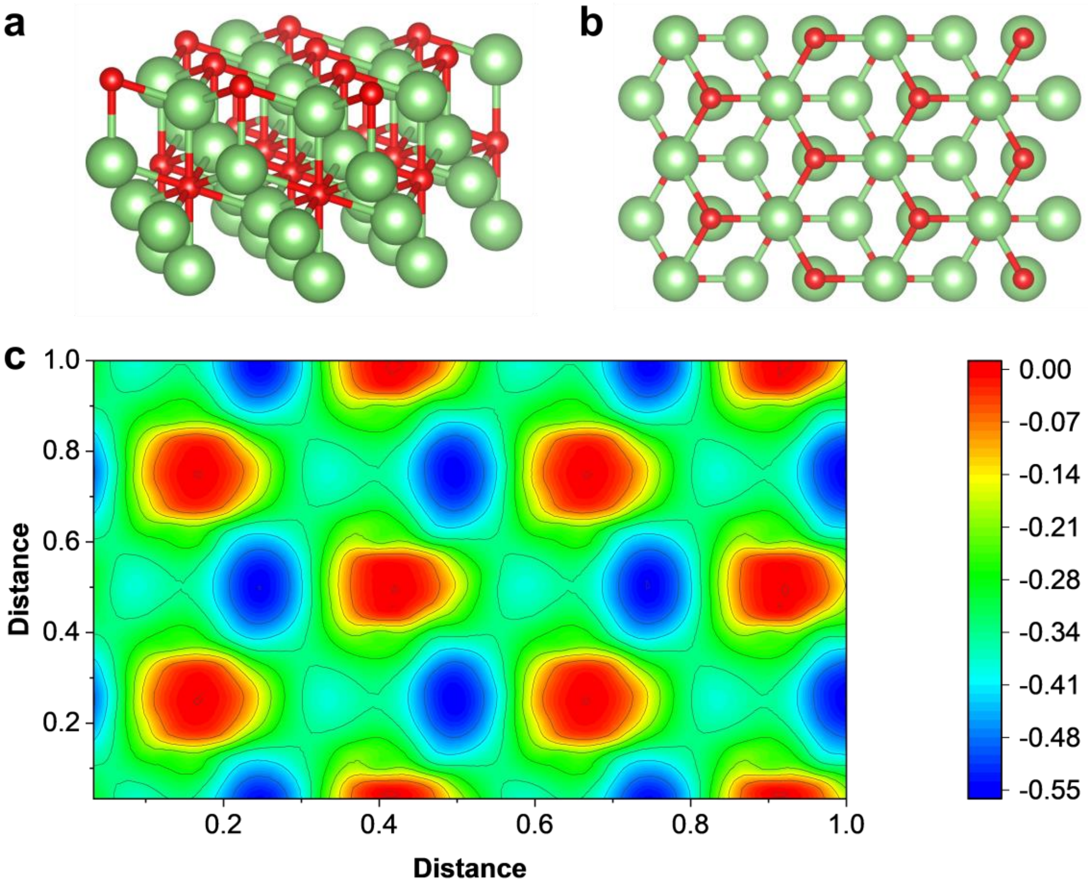

Figure S12. The configuration and binding energy landscape of Li_2O. (a) Li_2O configuration (Li: green balls, O: red balls), (b) (111) crystal facets and (c) binding energy landscape for Li^+ diffusion along grain boundaries. The unit of scale bar is eV.

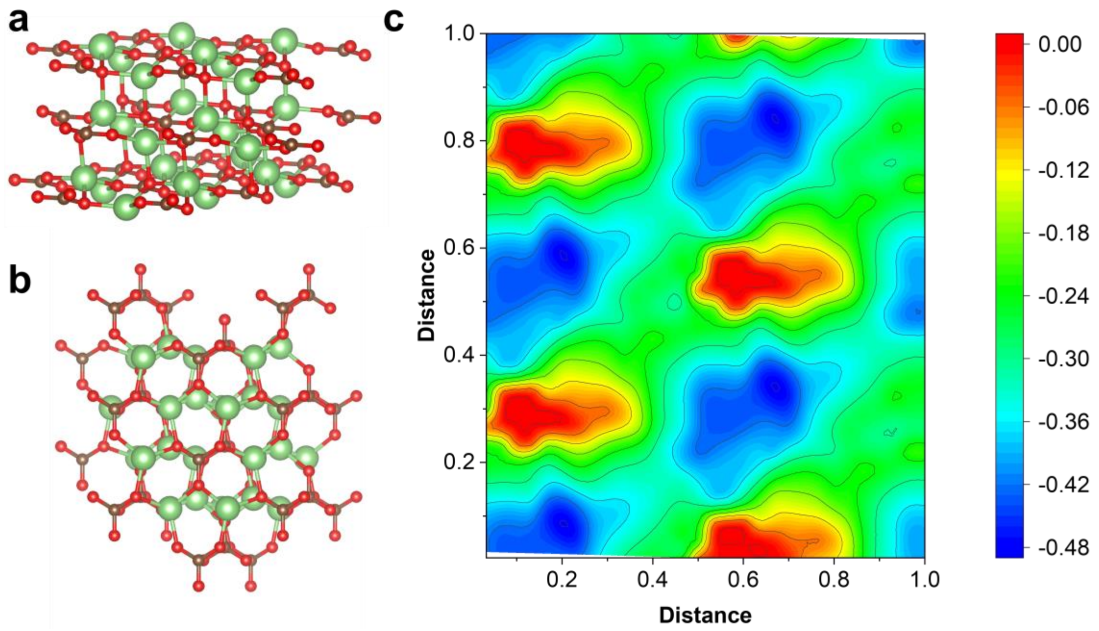

Figure S13. The configuration and binding energy landscape of Li_2CO_3. (a) Li_2CO_3 configuration (Li: green balls, O: red balls, C: brown balls), (b) (001) crystal facets and (c) binding energy landscape for Li^+ diffusion along grain boundaries. The unit of scale bar is eV.

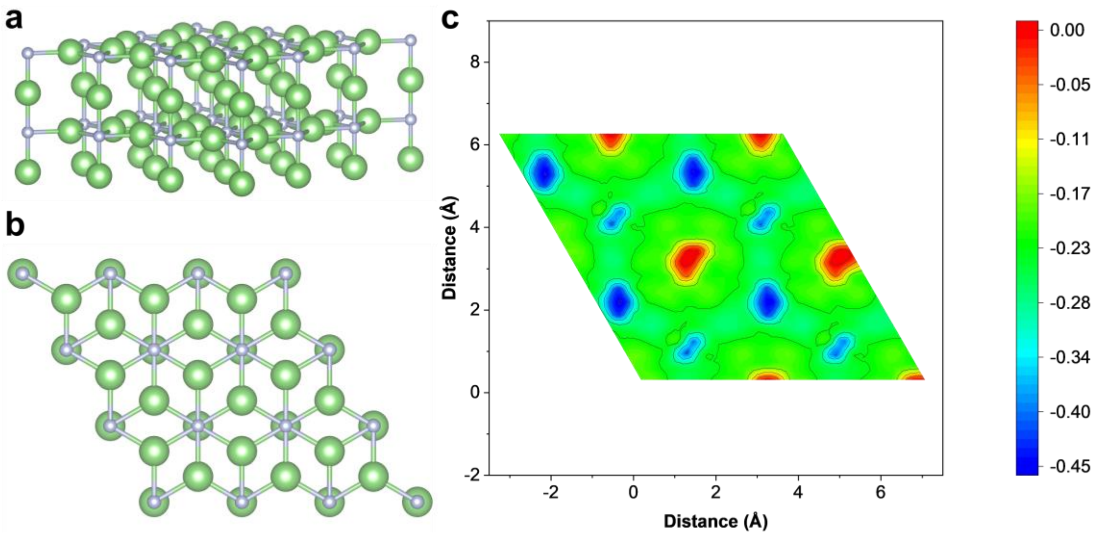

Figure S14. The configuration and binding energy landscape of α-Li_3N. (a) α-Li_3N configuration (Li: green balls, N: gray balls), (b) (001) crystal facets and (c) binding energy landscape for Li^+ diffusion along grain boundaries. The unit of scale bar is eV.

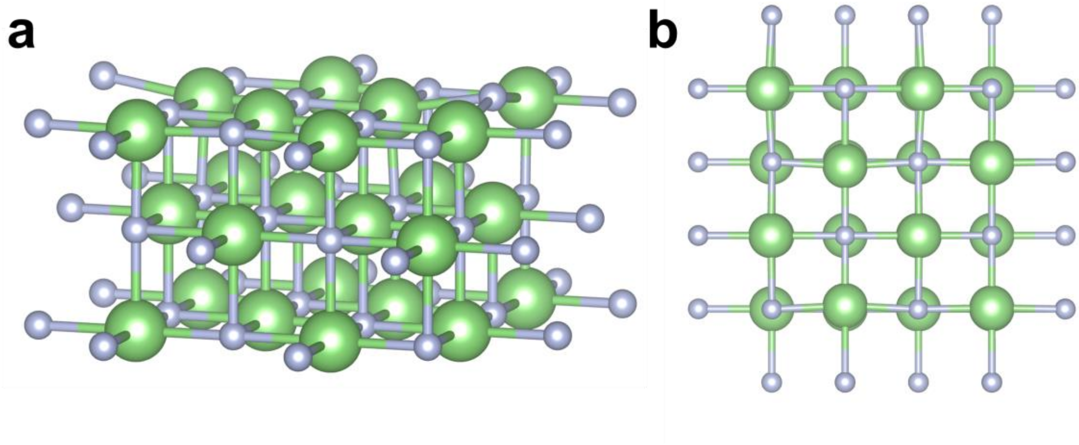

Figure S15. The configuration of LiF. (a) LiF configuration (Li: green balls, F: gray balls) and (b) (001) crystal facets.

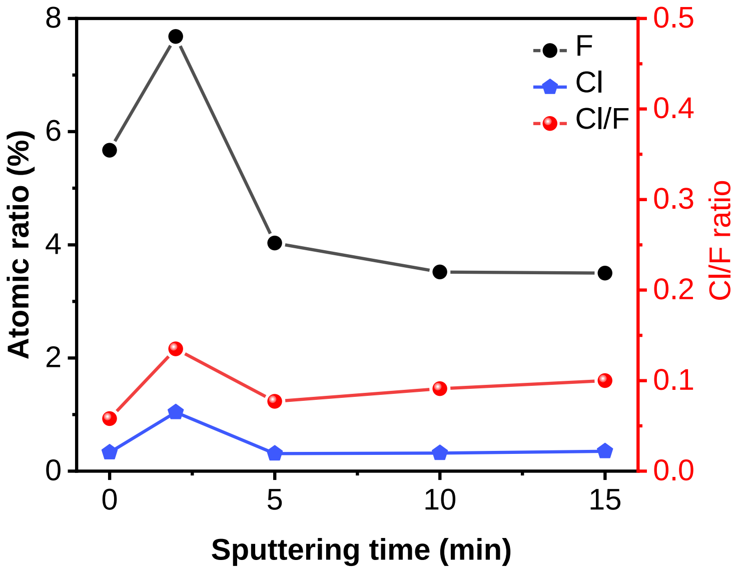

Figure S16. Atomic ratio of F and Cl in SEI with different sputtering time.

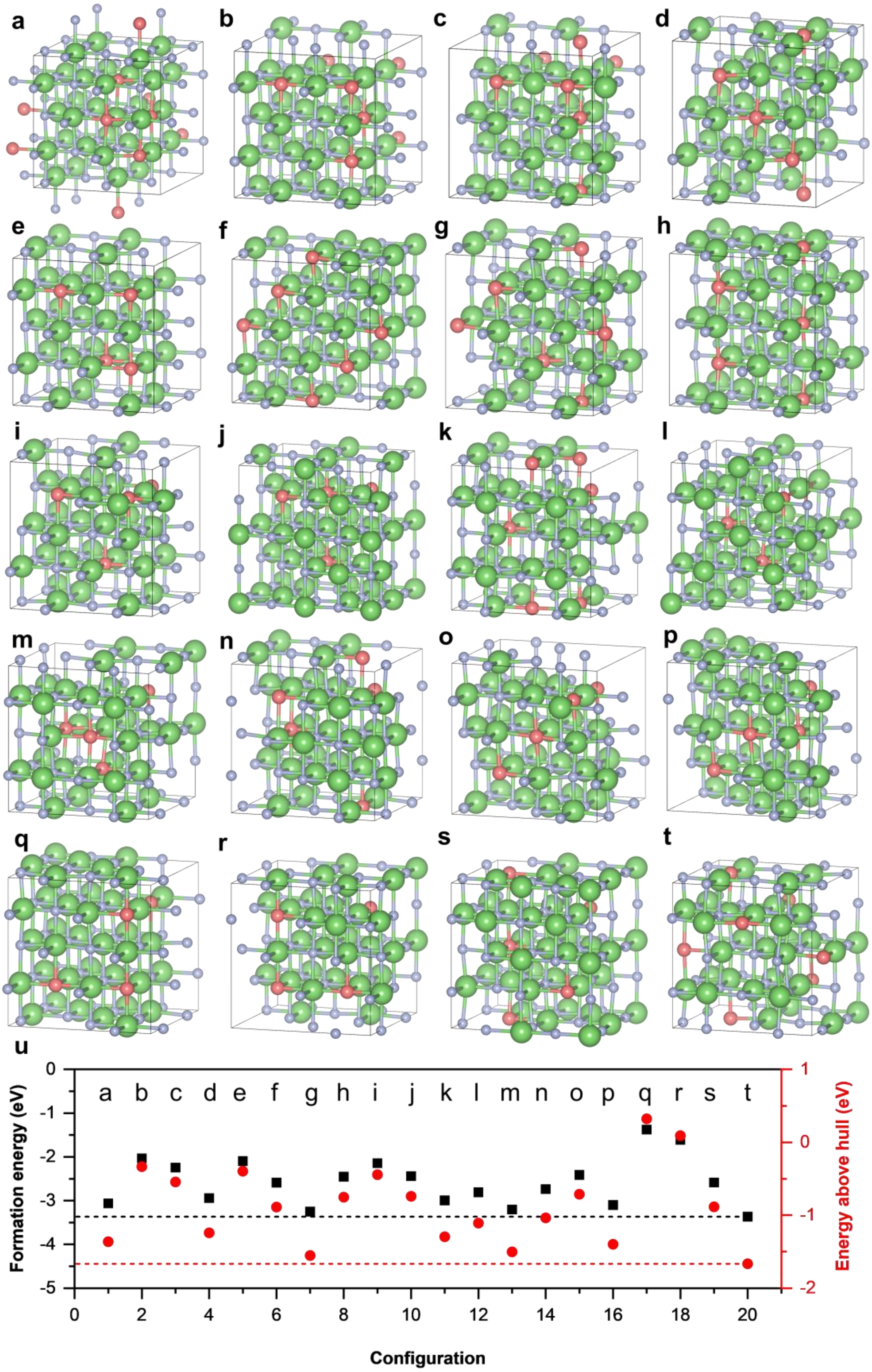

Figure S17. Comparison of different $LiF_{1-x}Cl_x$ configurations. (a-t) Possible configurations of $LiF_{1-x}Cl_x$ (Li: green balls, F: gray balls, Cl: red balls). (u) Formation energy and energy above hull of

each $LiF_{1-x}Cl_x$ configuration.

The thermodynamic feasibility of each $LiF_{1-x}Cl_x$ configuration was estimated by the formation energy and energy above hull (E_{hull}) in Figure S17u. E_{hull} of $LiF_{1-x}Cl_x$ is the relative formation energy (E_f) against segregation into thermodynamically stable phases of LiF and LiCl: $E_{hull} = E(LiF_{1-x}Cl_x) - (1-x)E(LiF)-xE(LiCl)$. The lowest E_f and E_{hull} both prove that the configuration in Figure S17t is the most stable state. This also demonstrates LiF and LiCl form a $LiF_{1-x}Cl_x$ crystal, other than a mixture or composite of $(1-x)$LiF-xLiCl.

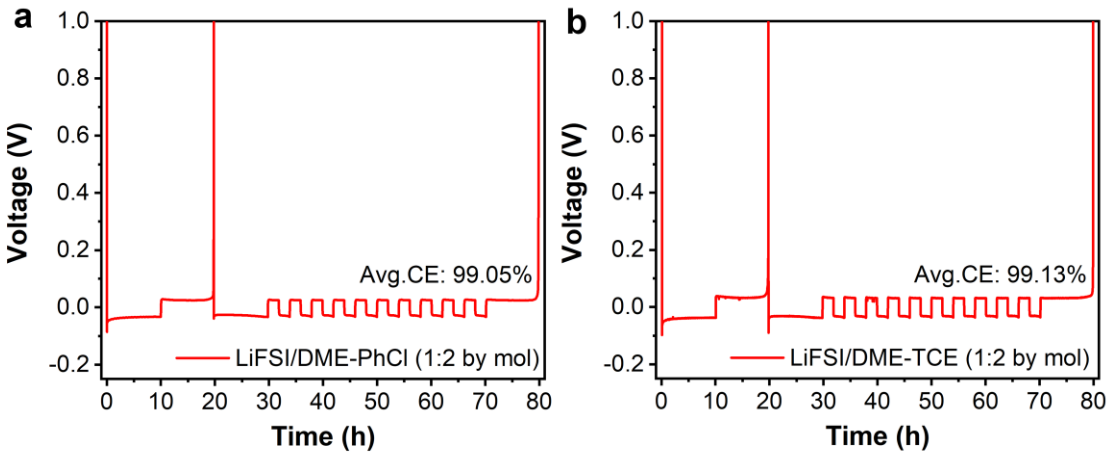

Figure S18. CE of Li||Cu cells with different electrolytes. (a) 2.5 M LiFSI/DME-PhCl and (b) 2.4 M LiFSI/DME-TCE.

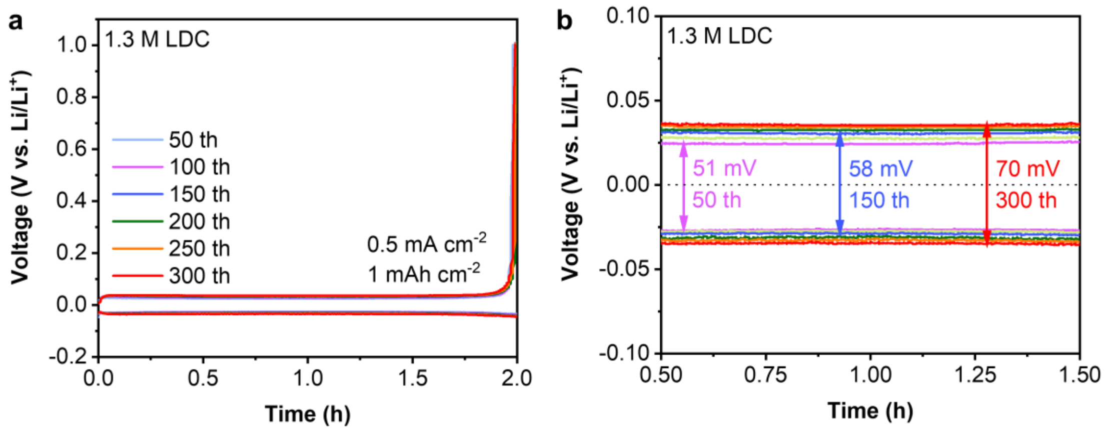

Figure S19. Overpotentials of Li||Cu cells in 1.3 M LiFSI/DME-DCE (1.3 M LDC) with different cycles. (a) The original and (b) amplified Voltage-time plots.

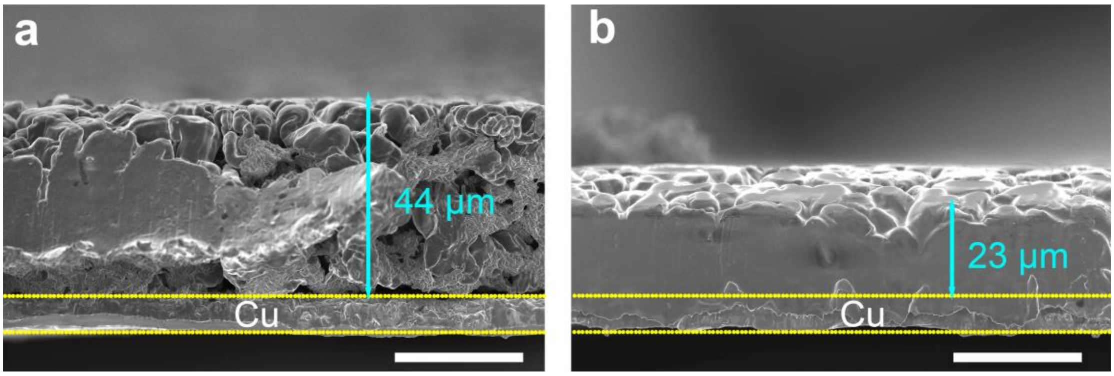

Figure S20. SEM images of the Li deposits. Cross section of Li deposited in (a) 6 M LiFSI/DME (HCE) and (b) 1.3 M LiFSI/DME-DCE (1.3 M LDC). The scale bar is 30 μm.

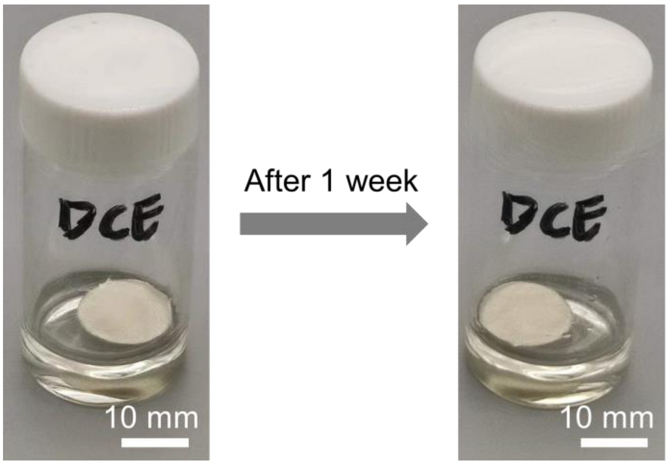

Figure S21. Comparison of optical images of a Li foil soaking in DCE for 1 week.

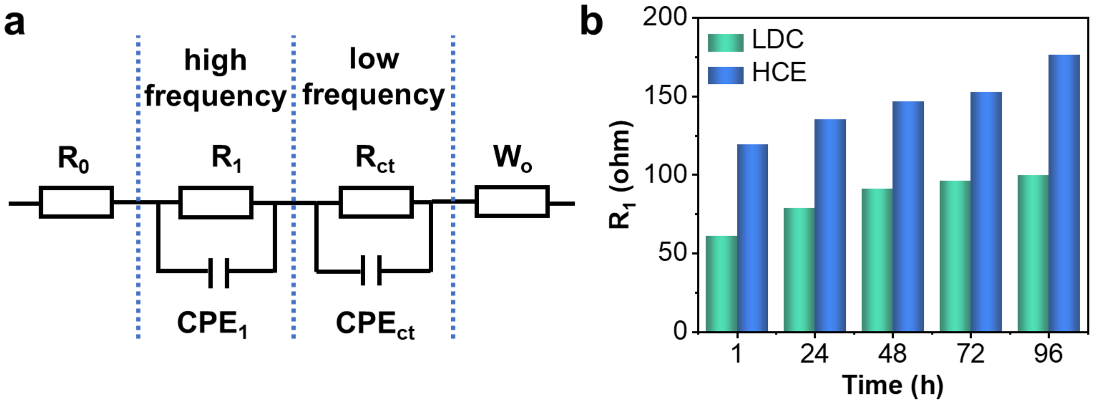

Figure S22. EIS tests of Li||Li cells. (a) The equivalent circuit of EIS plots in Figure 2h and (b) fitted R_1 values of Li||Li cells with 1.3 M LiFSI/DME-DCE (1.3 M LDC) and 6 M LiFSI/DME (HCE) electrolytes after different resting time.

In the equivalent circuit, R_0 is the ohmic resistance of the bulk electrolyte. In the high frequency region, R_1 and CPE_1 are the resistance of SEI layer and constant phase element. In the low frequency region, the R_{ct} and CPE_{ct} are the charge transfer resistance and constant phase element. W is the finite space (open) Warburg element that reflects the Li^+ diffusion through the solid[5].

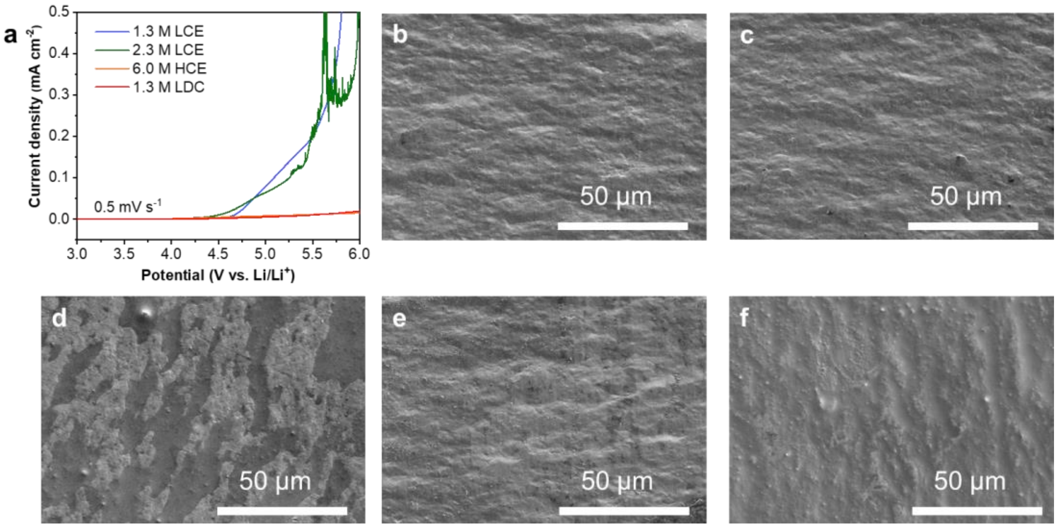

Figure S23. The corrosivity of different electrolytes to Al foils. (a) LSV curves of different electrolytes tested in Li||Al cells. SEM images of (b) original Al foils and Al foils held at 4.5 V for 12 h in (c) BE, (d) 1.3 M LCE, (e) HCE and (f) 1.3 M LDC electrolytes, respectively.

Li||Al cells were held at 4.5 V for 12 h to measure the corrosivity of different electrolytes to Al foils. Compared to the original Al foil (Figure S23b), BE electrolyte shows no corrosivity to the Al foil (Figure S23c), while the 1.3 M LCE obviously corrodes the Al foil due to the free FSI^- anions (Figure S23d). However, the Al corrosion is significantly inhibited in HCE (Figure S23e) and 1.3 M LDC electrolyte (Figure S23f).

Abbreviations:

1 M $LiPF_6$/EC-DMC (BE)

1.3 M LiFSI/DME (1.3 M LCE)

2.3 M LiFSI/DME (2.3 M LCE)

6 M LiFSI/DME (6 M HCE)

1.3 M LiFSI/DME-DCE (1.3 M LDC)

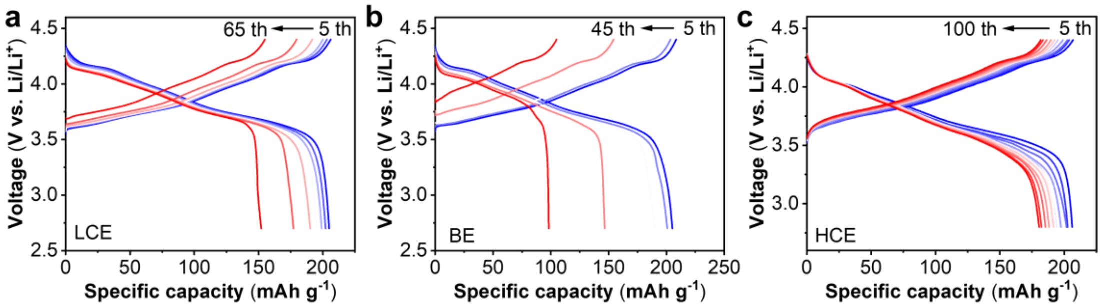

Figure S24. Voltage profiles of LMBs. Li||NCM811 cells with (a) 1.3 M LiFSI/DME (1.3 M LCE), (b) 1 M $LiPF_6$/EC-DMC (BE) and (c) 6 M LiFSI/DME (6 M HCE) electrolytes.

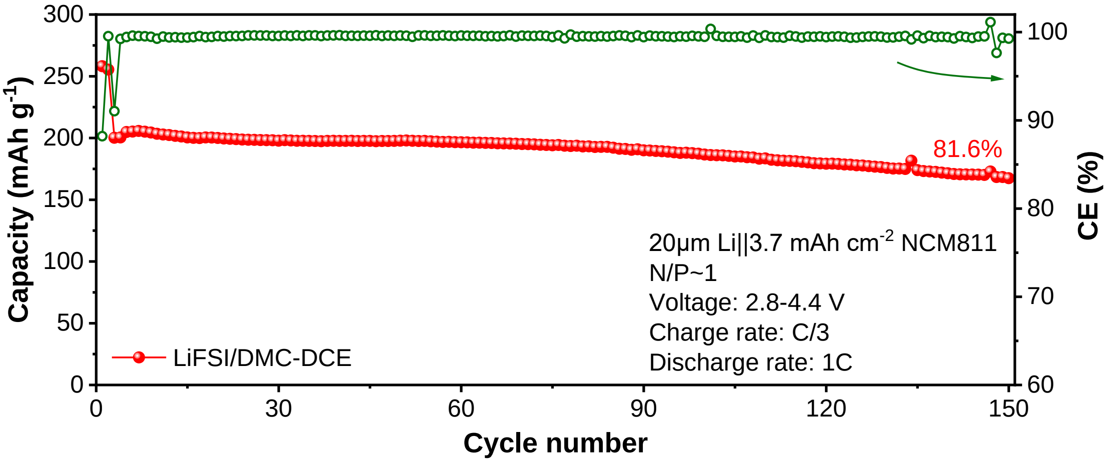

Figure S25. Cycle performance of Li||NCM811 cells tested in 2.2 M LiFSI/DMC-DCE electrolyte.

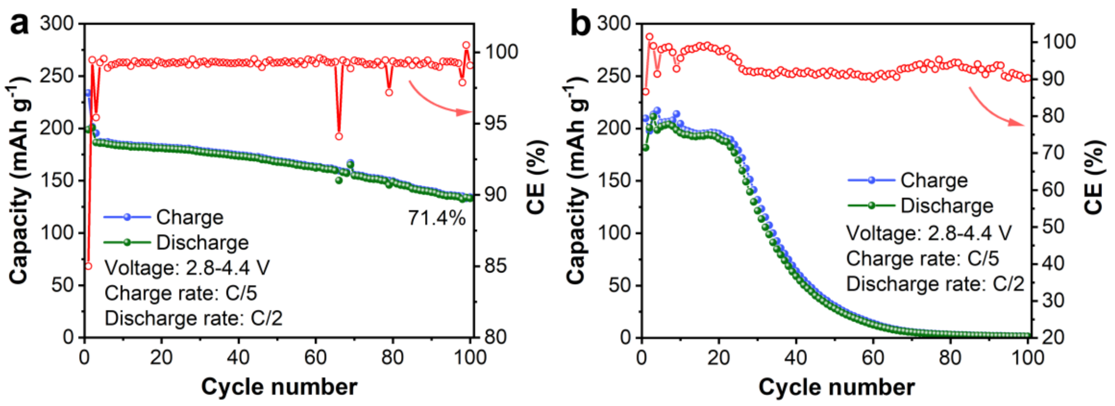

Figure S26. Cycle performance of Li||NCM811 cells. Cyle tests in (a) 2.5 M LiFSI/DME-PhCl and (b) 2.4 M LiFSI/DME-TCE. The anode is 20 μm Li and the cathode is 3.7 mAh cm^{-2} NCM811.

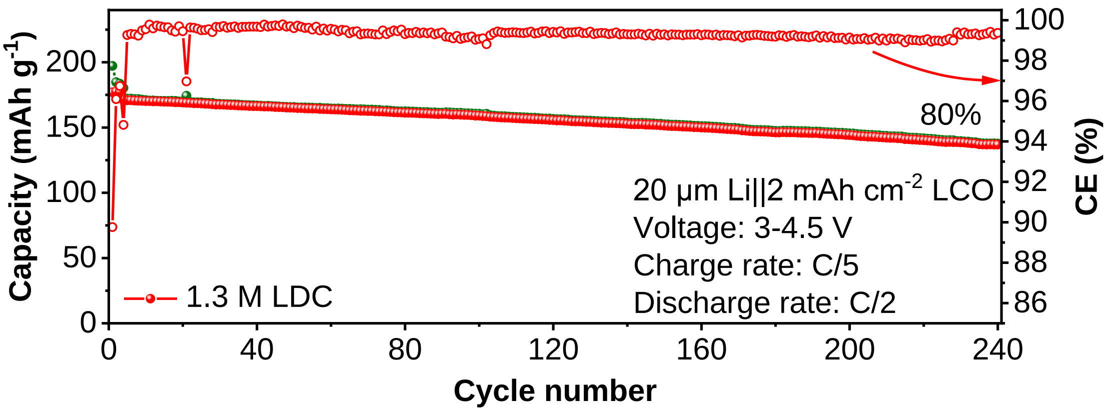

Figure S27. Cycle performance of Li||LCO batteries with 1.3 M LiFSI/DME-DCE (1.3 M LDC) electrolyte (1 C = 180 mA g^{-1}).

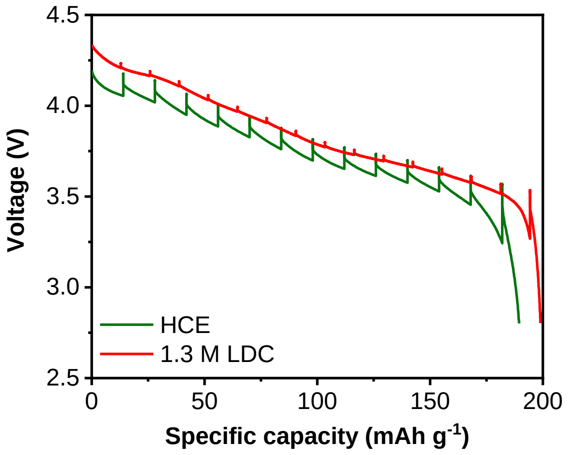

Figure S28. GITT curves of NCM811 in 6 M LiFSI/DME (6 M HCE) and 1.3 M LiFSI/DME-DCE (1.3 M LDC).

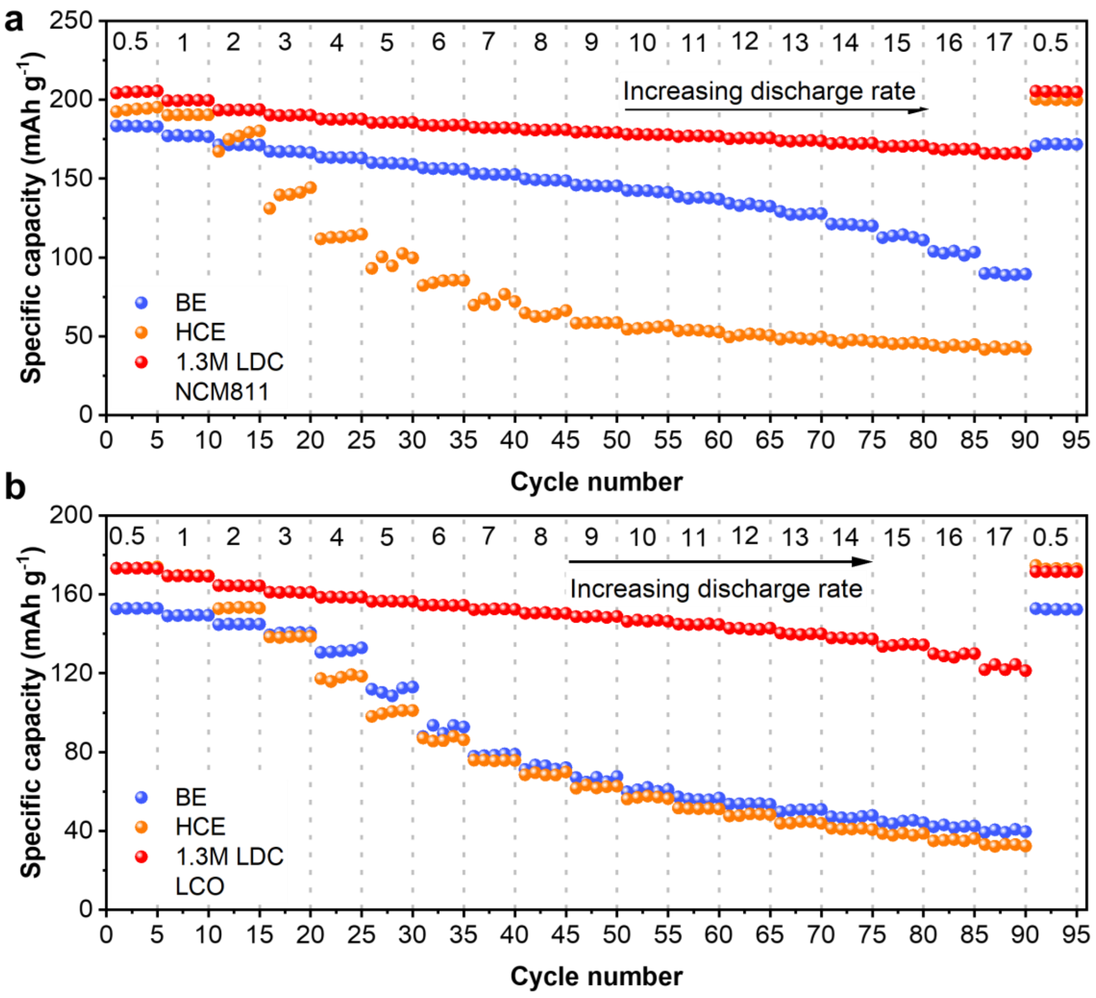

Figure S29. Rate capabilities of different electrolytes. (a) Li||NCM811 and (b) Li||LCO in 1 M $LiPF_6$/EC-DMC (BE), 6 M LiFSI/DME (6 M HCE) and 1.3 M LiFSI/DME-DCE (1.3 M LDC).

In 1.3 M LDC electrolyte, considerable capacities of NCM811 (166 mAh g^{-1}) and LCO (123 mAh g^{-1}) can be retained even at the high rate of 17 C and the specific capacity restores to the initial value when the current density returns to 0.5 C.

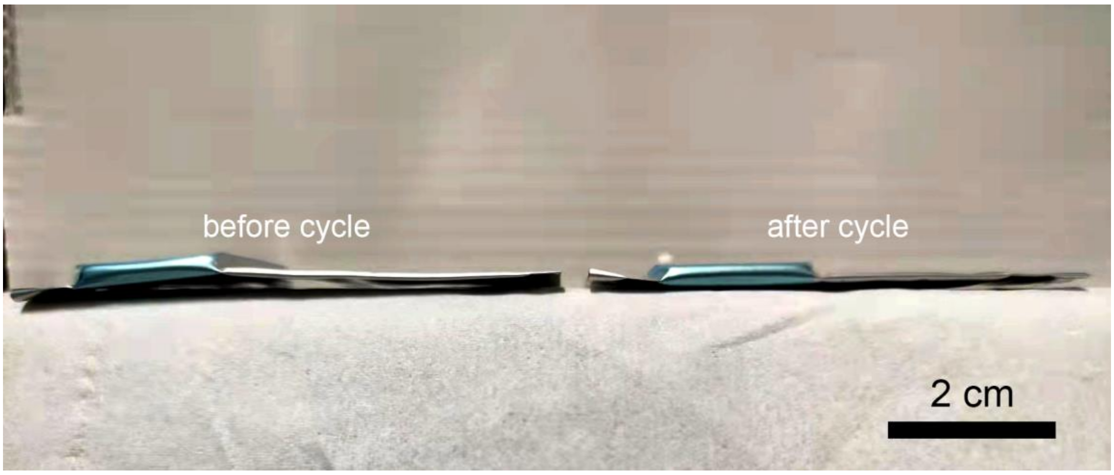

Figure S30. Comparison of anode-free pouch cells with 1.3 M LiFSI/DME-DCE (1.3 M LDC) before cycle and after cycle.

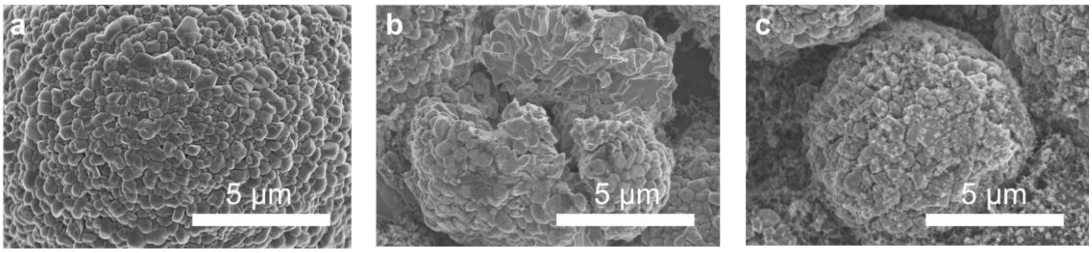

Figure S31. SEM images of NCM811 particles. (a) Pristine NCM811, (b) NCM811 cycled in 1 M $LiPF_6$/EC-DMC (BE) and (c) NCM811 cycled in 1.3 M LiFSI/DME-DCE (1.3 M LDC).

For NCM811 cycled in BE electrolyte, the significant fracture separates the electrical contacts among primary particles inducing more side reactions at the exposed surface[6, 7]. In sharp contrast, the primary particles remain intact in 1.3 M LDC electrolyte.

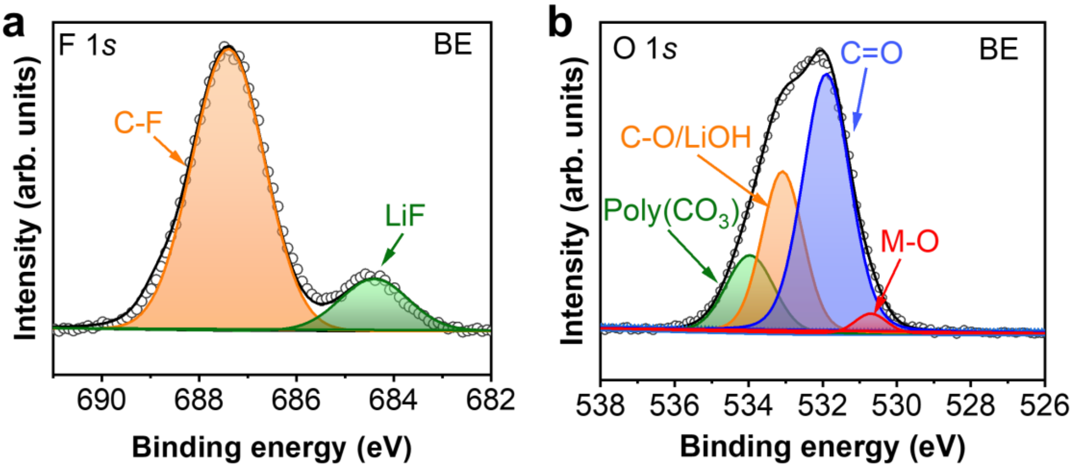

Figure S32. Surface chemistry of NCM811 cycled in 1 M $LiPF_6$/EC-DMC (BE) electrolyte: (a) F 1*s* and (b) O 1*s* spectra.

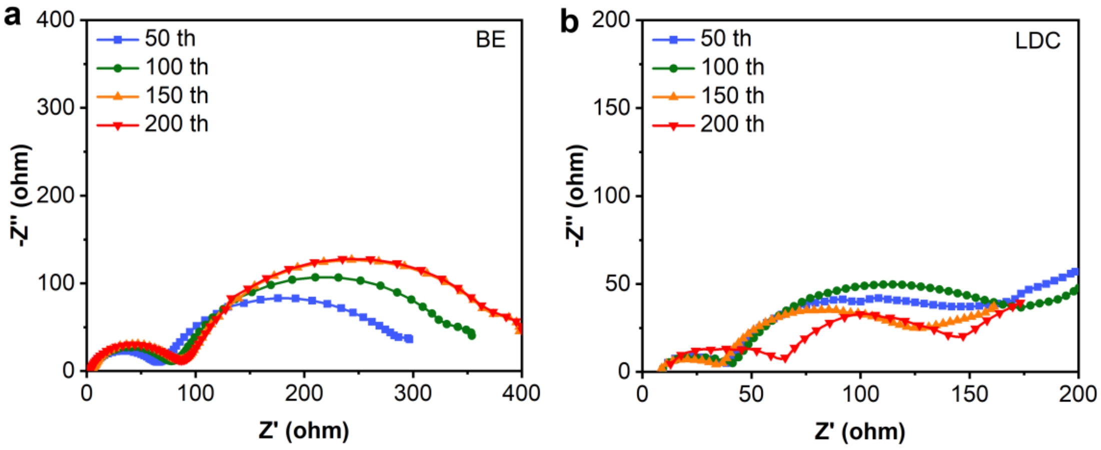

Figure S33. Evolution of impedance in Li||NCM811 cells. EIS plots of Li||NCM811 cells in (a) 1 M $LiPF_6$/EC-DMC (BE) and (b) 1.3 M LiFSI/DME-DCE (1.3 M LDC) electrolytes.

References

1. Qian J, Henderson WA, Xu W, Bhattacharya P, Engelhard M, Borodin O*, et al.* High rate and stable cycling of lithium metal anode. *Nat. Commun.* 2015, **6**(1)**:** 6362.

2. Ren X, Zou L, Cao X, Engelhard MH, Liu W, Burton SD*, et al.* Enabling high-voltage lithium-metal batteries under practical conditions. *Joule* 2019, **3**(7)**:** 1662-1676.

3. Piao N, Ji X, Xu H, Fan X, Chen L, Liu S*, et al.* Countersolvent electrolytes for lithium-metal batteries. *Adv. Energy Mater.* 2020, **10**(10)**:** 1903568.

4. Jiang Z, Zeng Z, Liang X, Yang L, Hu W, Zhang C*, et al.* Fluorobenzene, a low-density, economical, and bifunctional hydrocarbon cosolvent for practical lithium metal batteries. *Adv. Funct. Mater.* 2020, **31**(1)**:** 2005991.

5. Tatara R, Karayaylali P, Yu Y, Zhang Y, Giordano L, Maglia F*, et al.* The effect of electrode-electrolyte interface on the electrochemical impedance spectra for positive electrode in Li-ion battery. *J. Electrochem. Soc.* 2018, **166**(3)**:** A5090-A5098.

6. Ma L, Nie M, Xia J, Dahn JR. A systematic study on the reactivity of different grades of charged $Li[Ni_xMn_yCo_z]O_2$ with electrolyte at elevated temperatures using accelerating rate calorimetry. *J. Power Sources* 2016, **327:** 145-150.

7. Laszczynski N, Solchenbach S, Gasteiger HA, Lucht BL. Understanding electrolyte decomposition of graphite/NCM811 cells at elevated operating voltage. *J. Electrochem. Soc.* 2019, **166**(10)**:** A1853-A1859.
